# Three-dimensional motion corrected free-breathing simultaneous multislice-balanced steady state free precession myocardium perfusion imaging

**DOI:** 10.1016/j.jocmr.2025.101897

**Published:** 2025-04-21

**Authors:** Naledi Adam, Ronald Mooiweer, Andrew Tyler, Karl Kunze, Radhouene Neji, Peter Speier, Daniel Stäb, John Ng, Shino Kuriakose, Reza Razavi, Muhummad Sohaib Nazir, Amedeo Chiribiri, Sébastien Roujol

**Affiliations:** a School of Biomedical Engineering and Imaging Sciences, Faculty of Life Sciences and Medicine, King's College London, London, UK; b MR Research Collaborations, Siemens Healthcare Limited, Camberley, UK; c Cardiovascular predevelopment, Siemens Healthcare GmbH, Erlangen, Germany; d MR Research Collaborations, Siemens Healthcare Limited, Melbourne, Australia

**Keywords:** Myocardium perfusion imaging, Simultaneous multi-slice, Free-breathing, Prospective, Motion correction, Full left ventricular coverage

## Abstract

**Background:**

To develop a 3D motion-corrected simultaneous multislice-balanced steady state free precession (SMS)-bSSFP acquisition to enable free-breathing myocardial perfusion with high spatial resolution and coverage.

**Methods:**

A fast diaphragmatic respiratory navigator (fastNAV) module (<15 ms) was implemented into an SMS-bSSFP sequence for prospective slice-tracking. The remaining 2D in-plane motion was corrected using inline image registration. This approach (SMS-fastNAV) was compared to a reference SMS perfusion with 2D in-plane motion correction only (SMS-Ref) in 10 patients at 1.5T. Each subject underwent both perfusion protocols (six slices, resolution: 1.9 × 1.9 mm^2^) in a random order. The residual motion of the left ventricule (LV) was assessed by measuring the average DICE coefficient of the LV (avDICE) and the average displacement of the LV center of mass location (avCOM). Subjective assessment of image quality was also performed.

**Results:**

SMS-fastNAV led to lower residual LV motion than SMS-Ref before non-rigid image registration as shown by a higher avDICE (0.93±0.02 vs. 0.89±0.04, p<0.002) and decreased avCOM (2.82±0.89 mm vs. 4.23±1.29 mm, p = 0.005). After non-rigid image registration, SMS-fastNAV also led to higher avDICE score (0.95±0.01 vs. 0.94±0.02, p<0.027) and tended to decrease avCOM (0.97±0.21 mm vs. 1.01±0.25 mm, p = 0.23) with respect to SMS-Ref, suggesting a reduction in through-plane motion. There were no statistical significant differences between both approaches in terms of image quality (SMS-fastNAV: 1.79±0.50 vs. SMS-Ref: 2.00±0.59, p = 0.172).

**Conclusion:**

A 3D motion correction strategy was successfully developed for free-breathing SMS-bSSFP perfusion with high spatial coverage and resolution and provides improved motion correction with respect to standard in-plane image registration only.

## Graphical abstract

**Figure:**
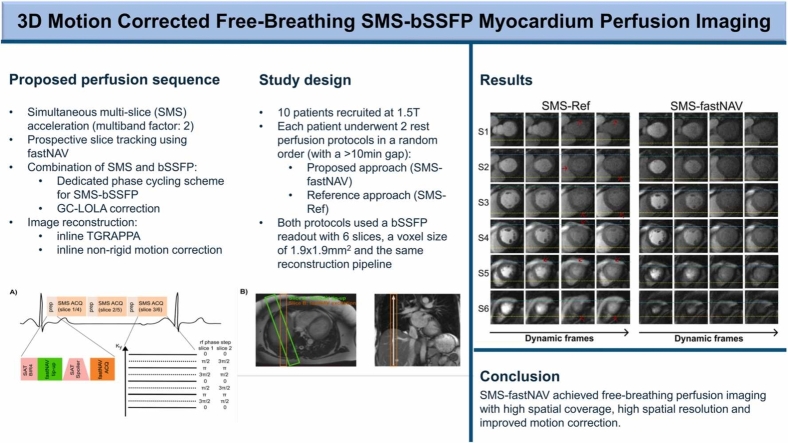

## 1. Introduction

Cardiovascular magnetic resonance imaging (CMR) using first-pass contrast-enhanced myocardial perfusion imaging is an established clinical technique for the assessment of hemodynamically significant coronary artery disease [1]. Standard clinical CMR perfusion protocols use a dynamic Electrocardiogram (ECG)-triggered saturation-recovery sequence, acquiring a limited 3–4 slices per heartbeat with an in-plane spatial resolution of 2–3 mm. Images are acquired during 60–90 s to cover the first-pass of the contrast agent, during which the patient is typically instructed to hold their breath. While different imaging readout have been evaluated, balanced steady state free precession (bSSFP) offers the highest signal to noise ratio (SNR) and CNR, which makes this approach attractive at 1.5T [2], [3].

CMR myocardial imaging with higher spatial coverage is desirable to improve assessment of ischemic burden [4], [5] which has important prognostic value [6]. Higher spatial coverage may also improve detection of apical perfusion defects [7], and assessment of ischemia and infarction when combined with late gadolinium enhancement [8], [9]. Higher in-plane spatial resolution (<2 × 2 mm^2^) is also desirable to reduce dark rim artifacts [10], [11], improve detection of subendocardial perfusion defect [8], [9], [12], [13], and assessment of transmural perfusion gradient [10], [11], [12], [13], [14]. While three-dimensional (3D) perfusion techniques have been proposed to increase spatial coverage, these techniques are commonly associated with a limited spatial resolution due to the short readout duration needed to minimize cardiac motion [15]. Simultaneous multi-slice (SMS) imaging [16], [17], [18], [19] has been proposed as an alternative for CMR perfusion imaging with high spatial coverage and resolution [20], [21], [22], [23], [24]. SMS uses multiband radiofrequency (RF) pulses to simultaneously excite multiple slices, which are then separated using parallel imaging reconstruction. SMS can be combined with bSSFP [25], [26] which has been successfully demonstrated for CMR perfusion imaging [20], [23], [24], [27].

Myocardial perfusion images are typically acquired during a single breath-hold covering the first-pass of the contrast agent. This adds complexity to the process by requiring precise synchronization of the breath-hold with the first pass of the contrast agent. Furthermore, this also requires that the patient can sustain a long breath-hold, which is not feasible for all patients. Long breath-holds can also cause changes to the heart rate which may cause images to be acquired at slightly different cardiac phases [28] and/or respiratory motion drift. Therefore, a free-breathing perfusion sequence is highly desirable to simplify the protocol and ensure feasibility in all patients but requires an efficient respiratory motion correction strategy.

Respiratory motion manifests itself as in-plane and through-plane motion of the myocardium. Most free-breathing perfusion sequences have addressed respiratory motion using retrospective image registration only, which allows for the correction of in-plane motion in reconstructed two-dimensional (2D) images [29], [30], [31], [32]. However, such a strategy does not correct for through-plane motion, potentially leading to inconsistent anatomical features over time, reduced robustness of in-plane image registration [33], and artifacts generated by advanced reconstruction techniques exploiting the temporal domain [34], [35].

Prospective slice-tracking techniques have been proposed for through-plane motion correction in myocardial perfusion sequences but remain challenging [33], [36], [37], [38]. A diaphragmatic navigator can be positioned before each saturation-recovery block, however, this may result in inconsistent signal over time due to varying saturation recovery times and insufficient quality due to the repeated saturation pulses [33]. The use of spatially selective saturation prepulses has been proposed to reduce the fluctuations of the diaphragmatic navigator signal, however, these pulses are typically associated with lower saturation efficiency [36], [37]. Alternatively, restoration of the magnetization in the diaphragm after each saturation pulse has been successfully demonstrated in this context [38], [39], with the fast navigator (fastNAV) technique offering reduced temporal footprint [39], which may be valuable to maximize the available time for perfusion data acquisition.

In this study, we developed a free-breathing myocardial perfusion sequence using SMS-bSSFP for high spatial coverage, resolution, and SNR/CNR, which was combined with prospective slice tracking using fastNAV and retrospective non-rigid image registration to enable efficient 3D motion correction. This approach is compared to a reference SMS-bSSFP sequence with retrospective non-rigid image registration (i.e., 2D in-plane motion correction only) in ten patients who underwent two rest free-breathing CMR perfusion imaging with each of the two techniques.

## 2. Methods

### 2.1 Proposed myocardial perfusion imaging sequence

**Fig. 1:**
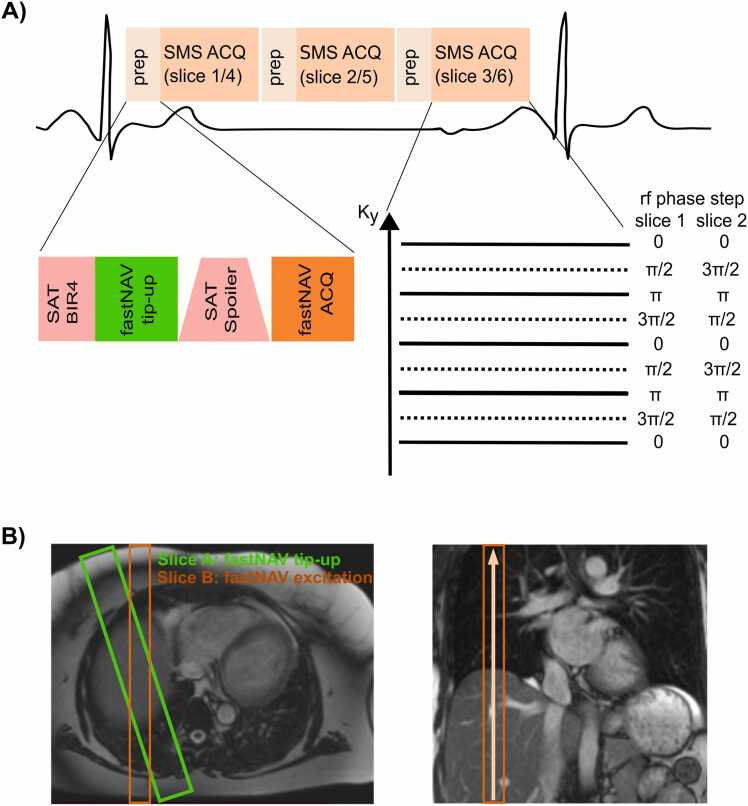
Diagram of the proposed free breathing SMS-bSSFP myocardial perfusion sequence with prospective slice-tracking using fast navigator (fastNAV). *SMS-bSSFP* simultaneous multislice-balanced steady state free precession, *ACQ* acquisition. *rf* radiofrequency, *SAT* saturation. *prep* preparation, *BIR4* B1-insensitive rotation 4

The proposed research sequence is depicted in Fig. 1A. Three saturation recovery blocks are used to acquire six slices per heartbeat. Each saturation recovery block consists of a saturation pre-pulse followed by the fastNAV acquisition and an imaging readout using SMS enabling the acquisition of two slices. Prospective slice-tracking of the SMS slices is achieved using the fastNAV signal. Images are reconstructed using temporal generalized autocalibrating partially parallel acquisition (TGRAPPA), image registration, and temporal filtering. The different elements of this research sequence are described below.

### 2.2 SMS-bSSFP imaging

SMS is based on multiband radiofrequency pulses. A multiband factor of 2 is used to simultaneously excite two slices. For improved slice separation during the reconstruction process, SMS is combined with controlled aliasing in parallel imaging results in higher acceleration (CAIPIRINHA) encoding which introduces a shift of the slices with respect to each other [40]. This is achieved using slice-dependent RF phase cycling which creates a phase ramp in k-space which, according to the Fourier shift theorem, leads to a shift in image space [40]. A shift of half of the field of view in the phase encoding direction is implemented, which is optimal for SMS with a multiband factor of 2.

The combination of SMS excitation, CAIPIRINHA encoding and bSSFP imaging requires to maintain the slice-specific k-space phase modulation necessary for CAIPIRINHA encoding and phase cycling requirements of bSSFP. This is addressed using the solution proposed by Stäb et al. [25] where a phase increment of π/2 and - π/2 is applied to successive RF pulses, leading to the following RF phase cycling scheme: [0, π/2, π, 3π/2 …] for slice #1 and [0, 3π/2, π, π/2 …] for slice #2. The use of independent RF phase cycling schemes for both bands results in a undesired slice-specific shift of the bSSFP frequency response, which is corrected using gradient-controlled local Larmour adjustment (GC-LOLA) [26]. The acceleration problem was reformulated from a 2D problem (2-fold slices and N-fold in-plane) to a one-dimensional (1D) problem (2×N-fold in-plane), with N the intended in-plane acceleration factor, as previously described [26]. This is achieved by prescribing a two-fold increased phase field-of-view (FOV), RF phase cycles distributing the two slices across the extended FOV, and an in-plane acceleration factor of 2×N. This approach thus enables the use of standard built in parallel imaging reconstruction methods where the shifted slice is reconstructed in the oversampled FOV and is ultimately separated by a simple FOV splitting operation.

### 2.3 FastNAV-based prospective slice tracking

FastNAV was implemented in the SMS-bSSFP sequence to enable prospective slice tracking in the feet-head direction [39]. FastNAV is applied within each saturation-recovery block and the fastNAV signal is acquired immediately before the SMS imaging readout to ensure consistent motion states between the respiratory navigator and imaging slices.

FastNAV is based on the prescription of two additional slices (slice A and B) intersecting on the dome of the diaphragm as seen in Fig. 1B. In a first step, the signal in slice A is restored during the saturation pulse. To this end, a B_1_-insensitive rotation 4 (BIR-4) 90° adiabatic saturation pulse is modified to include a slice selective tip-up pulse on slice A (−90°, 40 mm thickness) between the BIR-4 RF pulses and the spoiler gradient. The fastNAV signal is then acquired using a slice-selective excitation pulse on slice B (flip angle (FA) = 15°, 20 mm slice thickness) which predominantly corresponds to the signal (gradient echo) of the intersection profile of Slice A and B in foot-head direction. A delay of 5 ms after the fastNAV acquisition is inserted to allow for fastNAV reconstruction, diaphragmatic motion estimation, and real-time feedback to the acquisition sequence. The diaphragmatic motion is estimated using a standard inline cross-correlation analysis, as available from the vendor for conventional navigator signal. Prospective slice-tracking of the SMS imaging slices in the foot-head direction is finally performed using a tracking factor of 0.42 from Mooiweer et al. [39].

### 2.4 Image reconstruction

Images are first reconstructed using the inline TGRAPPA reconstruction. GRAPPA weights are calculated by combining data from multiple dynamics into a fully sampled k-space. Coil data are combined using sum of squares. This is followed by non-rigid image registration to correct for residual in-plane motion using the in-line solution that is commercially available from the manufacturer and based on [41]. Finally, a mild temporal filter (convolution with a 1D Gaussian kernel: standard deviation = 1.2) is applied to reduce noise contribution. All reconstructed steps were based on the inline implementation from the scanner manufacturer.

Additional images were reconstructed using only TGRAPPA reconstruction and sum-of-squares coil combination, to assess the impact of fastNAV without the application of in-plane motion correction.

### 2.5 In-vivo evaluation

Ten patients (7 M/3 F, age 59 ± 10 years, heartrate 69 ± 7 beats per min) referred for clinical CMR were recruited for this study. All patients were scanned on a 1.5T MRI scanner (MAGNETOM Aera; Siemens Healthineers AG, Erlangen, Germany) with a 32-element spine array coil and an 18-element body array coil. This study was approved by the National Research Ethics Service (15/NS/0030 and 23/NS/0034) and written informed consent was obtained from all patients.

To demonstrate the benefit of the proposed 3D motion correction, each patient underwent two rest free-breathing CMR perfusion protocols using the proposed sequence (SMS-fastNAV) and a similar one without fastNAV which served as reference (SMS-Ref). The two perfusion protocols were acquired within the same scan session, with a 10-minute gap between them to allow for the contrast agent to wash out. The order of the protocols was randomized across subjects. Each perfusion protocol used a dose of 0.075 mmol/kg gadobutrol (Gadovist, Bayer, Berlin, Germany). Both sequences used the same SMS bSSFP imaging parameters: TE/TR/FA: 1.24 ms/2.9 ms/50°, field of view: 360 × 326 mm^2^, spatial resolution 1.9 × 1.9 mm^2^, slice thickness: 10 mm, bandwidth: 1532 Hz/Px, 80 dynamics, slices: 6, in-plane acceleration factor: 3.5 with TGRAPPA undersampling scheme, SMS multiband factor: 2. Both sequences had an overall acceleration of 7, as previously validated [23], [27].

### 2.6 Quantitative assessment

To assess the performance of the proposed motion correction, the quantification of the temporal alignment of the left ventricle (LV) across all dynamics was assessed by measuring the average DICE coefficient of the LV (avDICE) [42] and the average displacement of the LV center of mass location (avCOM). Using our in-house MediaCare tool developed in MATLAB (The MathWorks, Natick, Massachusetts) [43], epicardial contours were manually drawn on temporal frames. This analysis was restrained to all dynamics where the epicardial contour of the myocardium was easily detectable (i.e., from peak enhancement in the myocardium until the end of the sequence). A higher avDICE and lower avCOM indicate improved LV registration.

To demonstrate the benefit of through-plane motion correction alone, avDICE and avCOM were measured for both sequences on the images reconstructed without in-plane motion correction, in one mid-ventricular slice. Since through-plane motion is difficult to quantify in 2D images, in-plane motion reduction is used as an indication of simultaneous reduction of through-plane motion. This is justified by the application of prospective slice-tracking in the foot-head direction which is expected to reduce both through-plane and in-plane motion jointly. These two metrics were then measured in all slices from both sequences after the entire reconstruction pipeline was applied. This analysis was also performed for different regions of the heart (base, mid, and apex).

To study the impact of the employed temporal filter, signal intensity temporal profiles obtained using the SMS-fastNAV sequence using the full reconstruction pipeline (with and without temporal filtering) were compared. This analysis was performed for the myocardial and blood curves using manually defined ROI in the septum and LV blood pool, respectively.

### 2.7 Qualitative assessment

Image quality was assessed by consensus of two experienced clinicians (AC and MSN with over 16- and 11-years' experience, respectively), blinded from the patients’ information and imaging sequence. Images were visualized using Radiant Image Viewer (Medixant, Poznan, Poland). Image quality was assessed using a 4-scale point system: 0 = poor and non-diagnostic, 1 = major artifact, but diagnostic, 2 = minor artifact, but diagnostic, 3 = excellent and diagnostic.

### 2.8 Statistical analysis

All results are reported as mean ± standard deviation. Normality of distribution was first assessed for continuous variables (avCOM and avDICE) using the Shapiro–Wilk test. Continuous variables with normal distribution were compared using the paired t test. The Wilcoxon signed-ranks test was used to compare continuous variables which were not normally distributed as well as the image quality scores. All statistical tests were 2-tailed. Statistical significance was defined as p<0.05.

## 3. Results

**Fig. 2:**
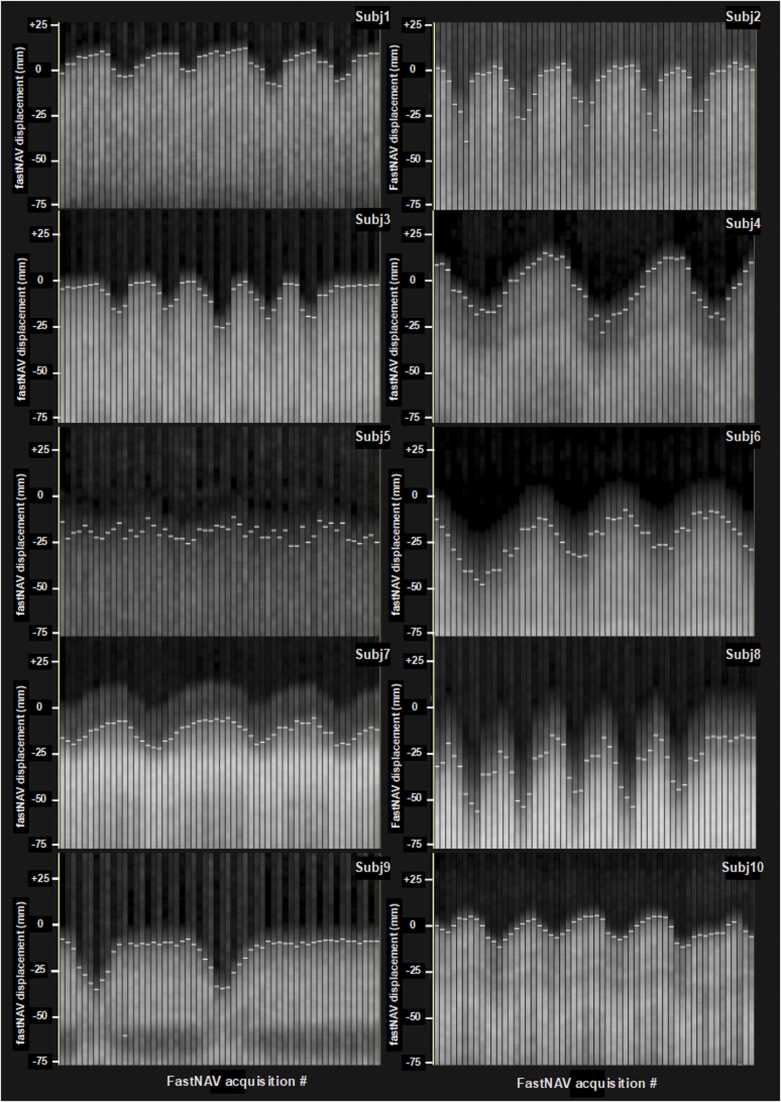
The fastNAV signal measured across all subjects (Subj1–10). The detected fastNAV positions are shown as white horizontal lines. Only the first 16 dynamics are shown, with three fastNAV acquisitions per heartbeat. *FastNAV* fast navigator.

Fig. 2 presents examples of the fastNAV tracking signal and position for all patients recruited in this study. The displayed fastNAV profiles correspond to the first 16 dynamics. Excellent signal consistency and SNR are observed over the temporal fastNAV profiles for most subjects. Lower SNR was, however, observed in one subject (S5), likely due to suboptimal navigator planning or incorrect positioning of the navigator in this patient. Overall, the fastNAV signal displacement distinctly shows the boundary between the diaphragm and the lungs, allowing the respiratory-induced diaphragmatic motion to be tracked.

**Fig. 3:**
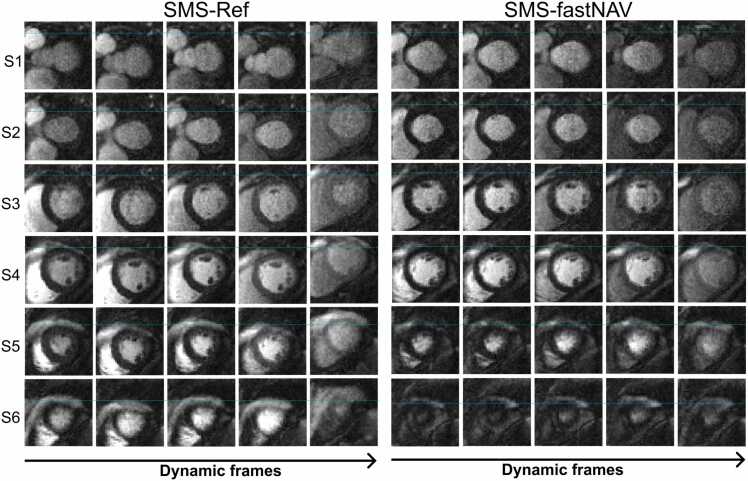
Perfusion images acquired in one patient (patient #4) using SMS-Ref and SMS-fastNAV, both reconstructed using TGRAPPA only (i.e., no in-plane motion correction). The same dynamics are shown for both techniques. SMS-fastNAV led to a reduction in the foot-head motion in all six slices. *SMS* simultaneous multi-slice, *FastNAV* fast navigator, *TGRAPPA* temporal generalized autocalibrating partial parallel acquisition, *Ref* reference.

**Fig. 4:**
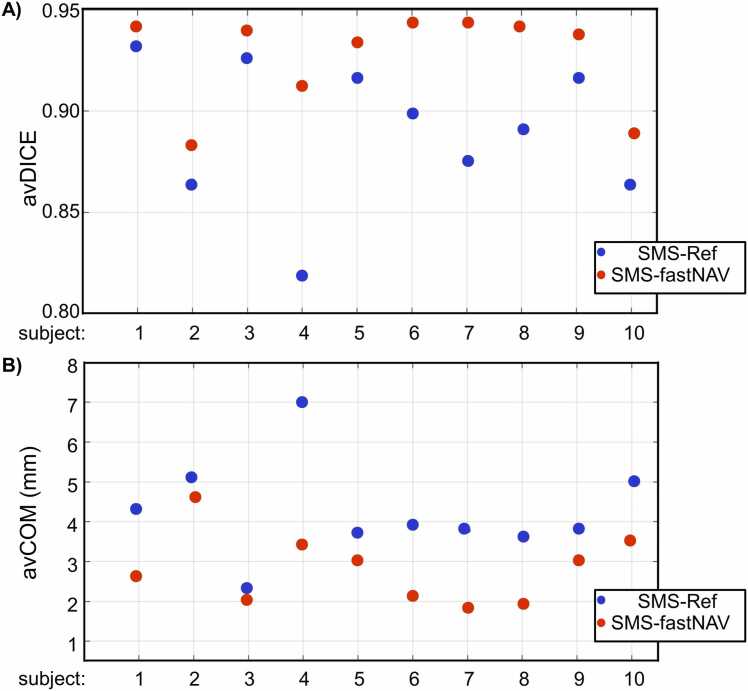
Quantitative comparison of residual motion in perfusion images acquired using SMS-Ref and SMS-fastNAV and reconstructed using TGRAPPA only (i.e., no in-plane motion correction). The avDICE (A) and avCOM (B) are reported from one mid-ventricular slice across all subjects. SMS-fastNAV provided improved avDICE and avCOM. *SMS* simultaneous multi-slice, *FastNAV* fast navigator, *TGRAPPA* temporal generalized autocalibrating partial parallel acquisition, *avDICE* average DICE coefficient, *avCOM* average DICE coefficient.

**Fig. 5:**
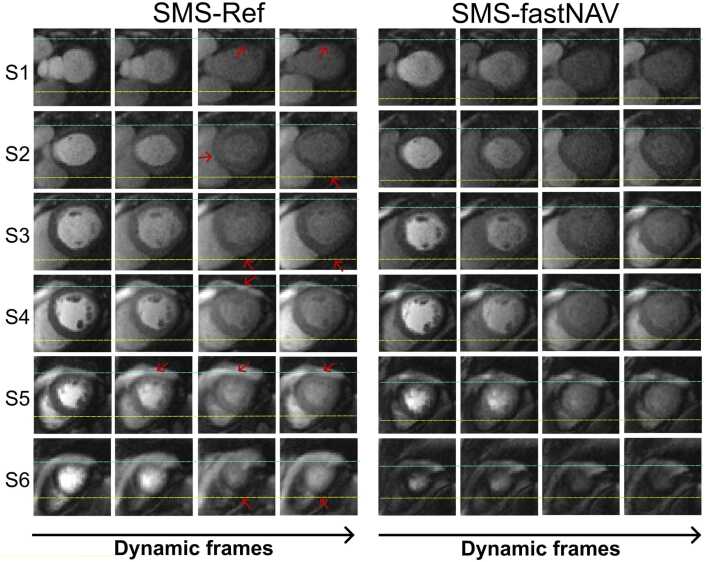
Comparison of SMS-Ref and SMS-fastNAV in one patient (patient #4), using the full reconstruction pipeline. The columns show four consecutive temporal points while the rows represent the six slices. Some residual motion is observed using SMS-Ref (shown by blue, yellow lines, and red arrows. SMS-fastNAV led to reduced through-plane motion which improved temporal consistency of the anatomy and reduced residual motion. *SMS* simultaneous multi-slice, *FastNAV* fast navigator, *Ref* reference.

Fig. 3 shows perfusion images from patient #4 acquired using SMS-fastNAV and SMS-Ref, both without retrospective image registration and temporal filtering. A video showing the entire first‐pass for both sequences is provided as Supplementary Information (Supplementary Information Video 1). Similar figures and videos obtained from two other patients (patients #7 and #8) are also provided in Supplementary information Fig. 1, Fig. 2 and Supplementary information Video 2 and 3. Native images acquired using fastNAV exhibit well reduced apparent motion of the myocardium with respect to SMS-Ref, illustrating the benefit of fastNAV to jointly reduce through-plane and in-plane motion. This is particularly visible in the most basal and apical slices which have inconsistent anatomical structures of the left ventricle over time using SMS-Ref. This effect was not observed with SMS-fastNAV. These findings were confirmed across all subjects as seen in Fig. 4 where SMS-fastNAV resulted in increased avDICE (0.93 ± 0.02 vs. 0.89 ± 0.04, p<0.002) and reduced avCOM (2.82 ± 0.89 mm vs. 4.23 ± 1.29 mm, p = 0.005), with respect to SMS-Ref.Example perfusion images acquired with both sequences in patient #4 and the entire reconstruction pipeline (including retrospective image registration and temporal filtering) are shown in Fig. 5. A video showing the entire first‐pass for both sequences is provided as Supplementary Information (Supplementary Information Video 4). Similar figures and videos obtained from the two other patients (patients #7 and #8) are also provided in Supplementary information Fig. 3, Fig. 4 and Supplementary information Video 5 and 6. Good image quality was obtained with both approaches. Ghosting artifacts can be seen in a few consecutive dynamics of the three most basal slices in the SMS-Ref data from Patient #4, which corresponded to a deep breathing event. No ghosting artifacts are observed in the corresponding SMS-fastNAV data for patient #4. No ghosting artifact is observed for both sequences in patients #6 and #7. Importantly, despite image registration, inconsistent anatomical information can be observed in the left ventricle over time using SMS-Ref, which was expected from the increase native through-plane motion. SMS-fastNAV provided improved temporal consistency of the left ventricle over time.

**Fig. 6:**
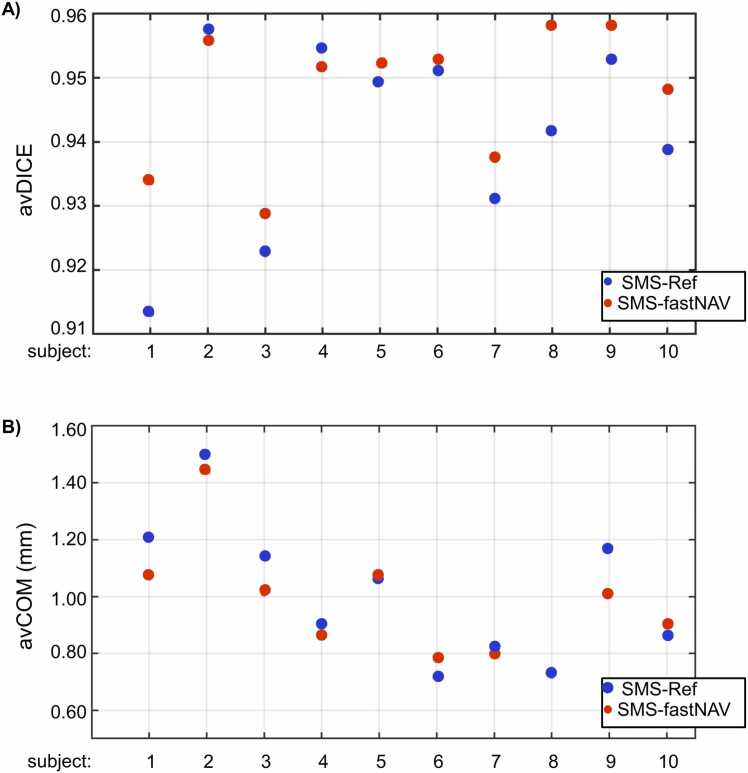
Quantitative comparison of residual motion in perfusion images acquired using SMS-Ref and SMS-fastNAV with the full reconstruction pipeline. The avDICE (A) and avCOM (B) were averaged over all slices and are shown for each subject and technique. SMS-fastNAV provided improved avDICE and avCOM. *SMS* simultaneous multi-slice, *FastNAV* fast navigator, *avDICE* average DICE coefficient, *avCOM* average DICE coefficient. *Ref* reference.

These results obtained with the entire reconstruction pipeline were confirmed across all subjects as shown in Fig. 6. Overall, SMS-fastNAV led to increased avDICE (0.95 ± 0.01 vs. 0.94 ± 0.02 for SMS-ref, p<0.027). The avCOM was reduced using SMS-fastNAV (0.97 ± 0.21 mm vs. 1.01 ± 0.25 mm for SMS-ref), although this difference did not reach statistical significance (p = 0.23). There were no statistically significant differences between SMS-fastNAV and SMS-Ref in terms of image quality (1.79 ± 0.50 vs. 2.00 ± 0.59, p = 0.172). These results were consistent for all regions of the heart, where SMS-fastNAV provided higher avDICE for the basal region (0.95 ± 0.01 vs. 0.94 ± 0.02, p = 0.02), mid-ventricular region (0.96 ± 0.01 vs. 0.95 ± 0.02, p = 0.04), and apical region (0.94 ± 0.01 vs. 0.93 ± 0.02, p = 0.11), although the difference for the apical region did not reach statistical significance. The avCOM was also consistently lower using SMS-fastNAV for the basal region (1.00 ± 0.25 vs. 1.07 ± 0.31, p = 0.24), mid-ventricular region (0.87 ± 0.18 vs. 0.99 ± 0.30, p = 0.05), and apical region (0.87 ± 0.27 vs. 0.90 ± 0.27, p = 0.56), although these differences did not reach statistical significance.

**Fig. 7:**
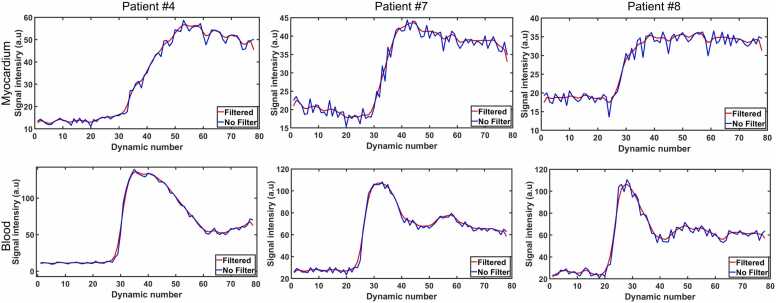
Signal intensity profiles measured in the septum (top) and LV blood pool (bottom) over all dynamics in three patients using SMS-fastNAV with the full reconstruction pipeline (“filtered”) and the full reconstruction pipeline without temporal filtering (“No filter”). The temporal filter substantially reduced the temporal variations without introducing a noticeable bias. *SMS* simultaneous multi-slice, *FastNAV* fast navigator, *LV* left ventricle/left ventricular.

The impact of the employed temporal filter is shown in Fig. 7. The signal intensity temporal profiles obtained using SMS-fastNAV sequence using the full reconstruction pipeline with and without temporal filter are shown in three patients. The temporal filter successfully reduced the temporal variations of the signal intensity profiles, without introducing noticeable bias in both the myocardium and the blood.

## 4. Discussion

A free-breathing SMS-bSSFP perfusion protocol including 3D motion correction was successfully developed. This approach enabled high spatial coverage, high spatial resolution, and improved motion correction, with no degradation of image quality. In comparison to the standard 2D motion correction approach, the proposed technique reduced both through-plane and in-plane motion in perfusion images, with improved temporal anatomical consistency of the left ventricle. The proposed 3D motion correction was implemented inline on the scanner.

FastNAV only adds 15 ms of time per saturation recovery block, thus having minimum impact on the timing of the perfusion sequence. Furthermore, it only requires the prescription of two intersecting slices on the dome of the right hemidiaphragm, which is identical to the standard commercially available cross-pair navigator provided by the vendor. Therefore, the small temporal footprint combined with the absence of required training to prescribe the navigator may facilitate the translation of this technology.

A fixed tracking factor was used in this study, which was shown to provide significant reduction of through-plane and in-plane motion across all patients. This is consistent with other clinical CMR protocols using navigator tracking which are also commonly based on a fixed tracking factor. However, the tracking factor is known to be subject-dependent [44]. Subject-specific tracking factor have been proposed for prospective slice tracking, but usually require an additional calibration scan [37], [39], [44]. Therefore, the benefit of a subject-specific tracking factor would need to outweigh the time invested to measure a subject-specific tracking factor, which will require further studies.

Image reconstruction was based on TGRAPPA combined with a mild temporal filter. The proposed 3D motion correction could also be combined with alternative reconstruction techniques. Iterative reconstruction techniques have been proposed as an alternative to parallel imaging alone in the context of SMS perfusion imaging [23], [45], to improve image quality and SNR [46]. However, these techniques usually exploit temporal regularization which lead to artifacts with the presence of respiratory motion [20], [23], [24], [27]. A variety of iterative reconstruction techniques with integrated motion correction have been proposed [47], [48], [49], [50], [51], [52], including for SMS perfusion imaging [45], and could be combined with the proposed strategy. However, these iterative techniques are associated with long reconstruction times, limiting their clinical applications. The use of deep-learning-based reconstruction may represent a promising approach to address this challenge [53], [54], [55]. Alternatively, the use of deep learning for image denoising has been proposed to better preserve temporal signal profiles and avoid temporal blurring which may arise from the temporal regularization of the aforementioned approaches [56]. Overall, the use of more advanced image reconstruction techniques may enable increased SMS multiband and in-plane accelerator factors which could enable further increased spatial coverage and resolution [20].

SMS was combined with bSSFP to maximize SNR/CNR, with CAIPIRINHA encoding achieved using slice-dependent RF phase cycling and GC-LOLA correction. Although GC-LOLA results in widening of bSSFP banding artifacts, no banding artifacts were observed in the left ventricle in this study, which is consistent with previous studies [20], [23], [24]. Blipped slice gradients can be used as an alternative to slice-dependent RF phase cycling for CAIPIRINHA encoding [57]. However, this approach may result in fat signal leakage due to the fat/water chemical shift causing an offset of the slice location of the excited fat signal compared to the water signal [20], [21], [22], [58]. Furthermore, the proposed approach could be implemented with any other imaging readout, such as gradient echo imaging, which may be particularly relevant if imaging at higher field strengths, such as 3T and above.

Ghosting artifacts could occasionally occur in the presence of deep breathing events. These are likely due to the TGRAPPA reconstruction combining multiple dynamics to derive a fully sampled k-space and calculates weights. As a result, these GRAPPA weights will be inaccurate for outlier breathing position, such as those during deep breathing events. Due to their low rate of occurrence, a larger cohort study will be necessary to characterize the sensitivity of these two SMS sequences to deep breathing events.

## 5. Limitations

This study has several limitations. First, no comparison with a standard 3 slices breath-hold perfusion protocol (i.e., no prospective slice tracking) was performed. It is difficult to perform more than 2 rest perfusion protocols in the same session and we used an SMS sequence with no prospective slice tracking for the following reason. We previously demonstrated the benefit of prospective slice tracking using fastNAV in a standard 3 slice protocol (in the absence of in-plane motion correction) [39]. We also demonstrated the benefit of SMS without prospective slice tracking to improve spatial coverage without degradation of spatial resolution and image quality, and sequence-related artifacts (including blurring and ghosting) with respect to a standard 3 slice protocol [23]. Therefore, building on these findings, the use of SMS with no prospective slice tracking appeared as a good strategy to demonstrate the value of the proposed technology with respect to existing state-of-the-art.

Secondly, the two perfusion protocols were separated by 10 min to allow for contrast washout, as previously recommended [59]. However, this usually does not allow for complete contrast washout and may have affected baseline images of the second scan. However, since the order of both approaches was randomized across subjects, the influence of this effect should have been kept to the minimum.

Thirdly, the proposed approach was only evaluated using rest perfusion protocols. However, with the small temporal footprint of fastNAV, the proposed approach can be acquired in less than 550 ms per heartbeat and is therefore compatible with stress conditions up to a heart rate of 110 beats per minute.

Finally, this study had a small sample size. The purpose of this study was to demonstrate the feasibility of 3D motion-corrected free-breathing SMS-bSSFP perfusion imaging. Evaluation of this technology at stress in a larger cohort of patients referred for assessment of coronary artery disease is now warranted.

## 6. Conclusion

SMS-bSSFP was successfully combined with a 3D motion correction strategy to enable free-breathing CMR perfusion imaging with high spatial coverage, high spatial resolution, and efficient through-plane and in-plane respiratory motion correction.

## Funding

Biomedical Research Centre at Guy's and St Thomas' National Health Service (NHS) Foundation Trust; British Heart Foundation (BHF), Grant/Award Numbers:(PG/19/11/34243), (PG/21/10539); Engineering and Physical Sciences Research Council (EPSRC), Grant/Award Number: (EP/R010935/1); King's College London; Innovate UK, Grant/Award Number: (68539); National Institute for Health Research (NIHR); The Government of Botswana; Wellcome Trust (WT 203148/Z/16/Z). The views expressed are those of the authors and not necessarily those of the NHS, the NIHR, nor the Department of Health. For the purpose of open access, the author has applied a CC BY public copyright licence to any Author Accepted Manuscript version arising from this submission.

## Author contributions

S.R. conceived the study. S.R., A.C., and N.A. designed the study. N.A., R.M., A.T., K.K., P.S., D.S. implemented the perfusion sequence. N.A., R.R., A.C., J.N., S.K. performed the experiments and data acquisition. N.A., M.S.N., A.C. performed the data analysis. N.A. and S.R. drafted the manuscript. All authors reviewed and gave critical feedback to the manuscript.

## Ethics approval and consent

All patient participants gave informed written consent for this study which had research ethics committee approval (approval number 15/NS/0030 and 23/NS/0034) and was in full compliance with the declaration of Helsinki.

## Consent for publication

Consent for publication was obtained from all participants in the study.

## Declaration of competing interests

The authors declare the following financial interests/personal relationships which may be considered as potential competing interests. Sebastien Roujol reports financial support and equipment, drugs, or supplies were provided by Biomedical Research Centre at Guy’s and St Thomas’ National Health Service. Sebastien Roujol reports financial support was provided by British Heart Foundation. Sebastien Roujol reports financial support was provided by Engineering and Physical Sciences Research Council. Sebastien Roujol reports financial support, administrative support, article publishing charges, and equipment, drugs, or supplies were provided by King’s College London. Sebastien Roujol reports financial support was provided by Innovate UK. Sebastien Roujol reports financial support was provided by National Institute for Health Research. Naledi Adam reports financial support was provided by Republic of Botswana Government. Sebastien Roujol reports financial support was provided by Wellcome Trust. Karl Kunze reports a relationship with Siemens Healthcare Limited that includes: employment. Peter Speier reports a relationship with Siemens Healthcare Limited that includes: employment. Daniel Stab reports a relationship with Siemens Healthcare Limited that includes: employment. KK, PS and DS are employees of Siemens Healthcare Limited. RM was seconded to Siemens Healthcare Limited. If there are other authors, they declare that they have no known competing financial interests or personal relationships that could have appeared to influence the work reported in this paper.

## Availability of data and materials

The datasets used and/or analyzed during the current study are available from the corresponding author on reasonable request.

## Acknowledgements

The authors would like to acknowledge the King’s and St. Thomas’ clinical staff who assisted with acquiring the patient scans.
