# Supplementary material for: Three-dimensional motion corrected free-breathing simultaneous multislice-balanced steady state free precession myocardium perfusion imaging
Source: J Cardiovasc Magn Reson. 2025 Apr 21;27(2):101897. doi: 10.1016/j.jocmr.2025.101897 (PMC12271901; doi:10.1016/j.jocmr.2025.101897)
Supplement: Supplementary file 1 — Supplementary material [file mmc1.docx]

**Supplementary Information** **Figure 1:** Perfusion images acquired in one patient (patient #7) using SMS-Ref and SMS-fastNAV, both reconstructed using TGRAPPA only (i.e. no in-plane motion correction). Large displacements of the myocardium are observed using SMS-Ref, which are well reduced using SMS-fastNAV.

**Supplementary Information** **Figure 2:** Perfusion images acquired in one patient (patient #8) using SMS-Ref and SMS-fastNAV, both reconstructed using TGRAPPA only (i.e. no in-plane motion correction). Large displacements of the myocardium are observed using SMS-Ref, which are well reduced using SMS-fastNAV.

**Supplementary Information** **Figure 3:** Comparison of SMS-Ref and SMS-fastNAV in one patient (patient #7), using the full reconstruction pipeline. SMS-fastNAV led to improved co-registration of the images.

**Supplementary Information** **Figure 4:** Comparison of SMS-Ref and SMS-fastNAV in one patient (patient #8), using the full reconstruction pipeline. SMS-fastNAV led to improved co-registration of the images.

**Supplementary Information Video 1:** Comparison of SMS-Ref (top) and SMS-fastNAV (bottom), both reconstructed using TGRAPPA only (i.e. no in-plane motion correction), in subject #4. A reduction in motion is seen across all 6 slices using SMS-fastNAV.

**Supplementary Information Video 2:** Comparison of SMS-Ref (top) and SMS-fastNAV (bottom), both reconstructed using TGRAPPA only (i.e. no in-plane motion correction), in subject #7. A reduction in motion is seen across all 6 slices using SMS-fastNAV.

**Supplementary Information** **Video 3:** Comparison of SMS-Ref (top) and SMS-fastNAV (bottom), both reconstructed using TGRAPPA only (i.e. no in-plane motion correction), in subject #8. A reduction in motion is seen across all 6 slices using SMS-fastNAV.

**Supplementary Information Video 4:** Comparison of SMS-Ref (top) and SMS-fastNAV (bottom), both with the full reconstruction pipeline, on subject # 4. SMS-fastNAV led to improved temporal alignment of images for all slices.

**Supplementary Information Video 5:** Comparison of SMS-Ref (top) and SMS-fastNAV (bottom), both with the full reconstruction pipeline, on subject # 7. SMS-fastNAV led to improved temporal alignment of images for all slices.

**Supplementary Information Video 6:** Comparison of SMS-Ref (top) and SMS-fastNAV (bottom), both with the full reconstruction pipeline, on subject # 8. SMS-fastNAV led to improved temporal alignment of images for all slices.
