# Supplementary figures and images for: Three-dimensional motion corrected free-breathing simultaneous multislice-balanced steady state free precession myocardium perfusion imaging
Source: J Cardiovasc Magn Reson. 2025 Apr 21;27(2):101897. doi: 10.1016/j.jocmr.2025.101897 (PMC12271901; doi:10.1016/j.jocmr.2025.101897)

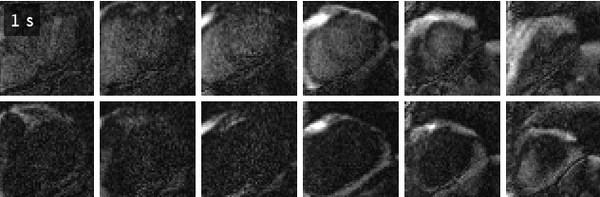

Supplement: Supplementary file 2 — Supplementary material [file mmc2.zip › SupplementaryVideo1.gif]

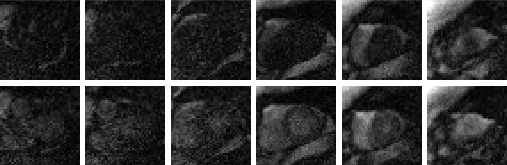

Supplement: Supplementary file 3 — Supplementary material [file mmc3.zip › SupplementaryVideo2.gif]

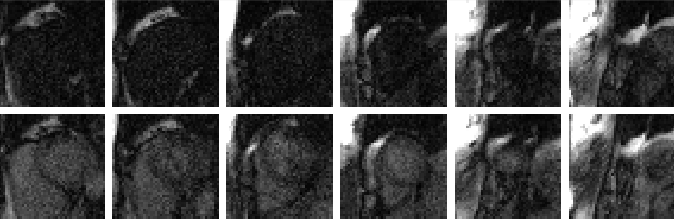

Supplement: Supplementary file 4 — Supplementary material [file mmc4.zip › SupplementaryVideo3.gif]

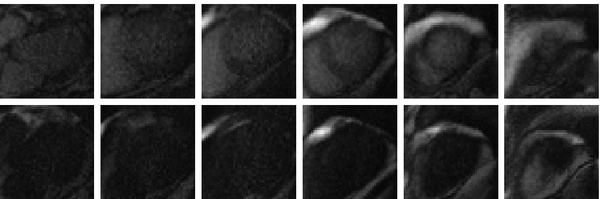

Supplement: Supplementary file 5 — Supplementary material [file mmc5.zip › SupplementaryVideo4.gif]

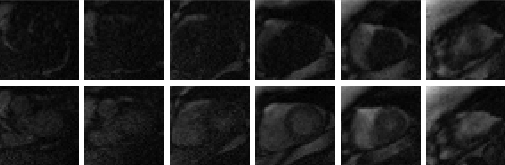

Supplement: Supplementary file 6 — Supplementary material [file mmc6.zip › SupplementaryVideo5.gif]

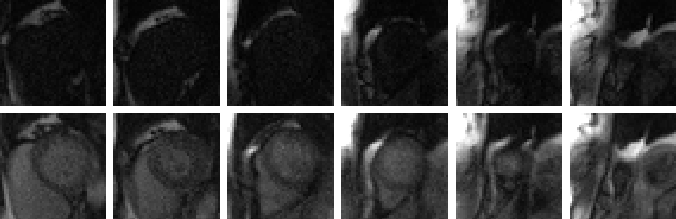

Supplement: Supplementary file 7 — Supplementary material [file mmc7.zip › SupplementaryVideo6.gif]

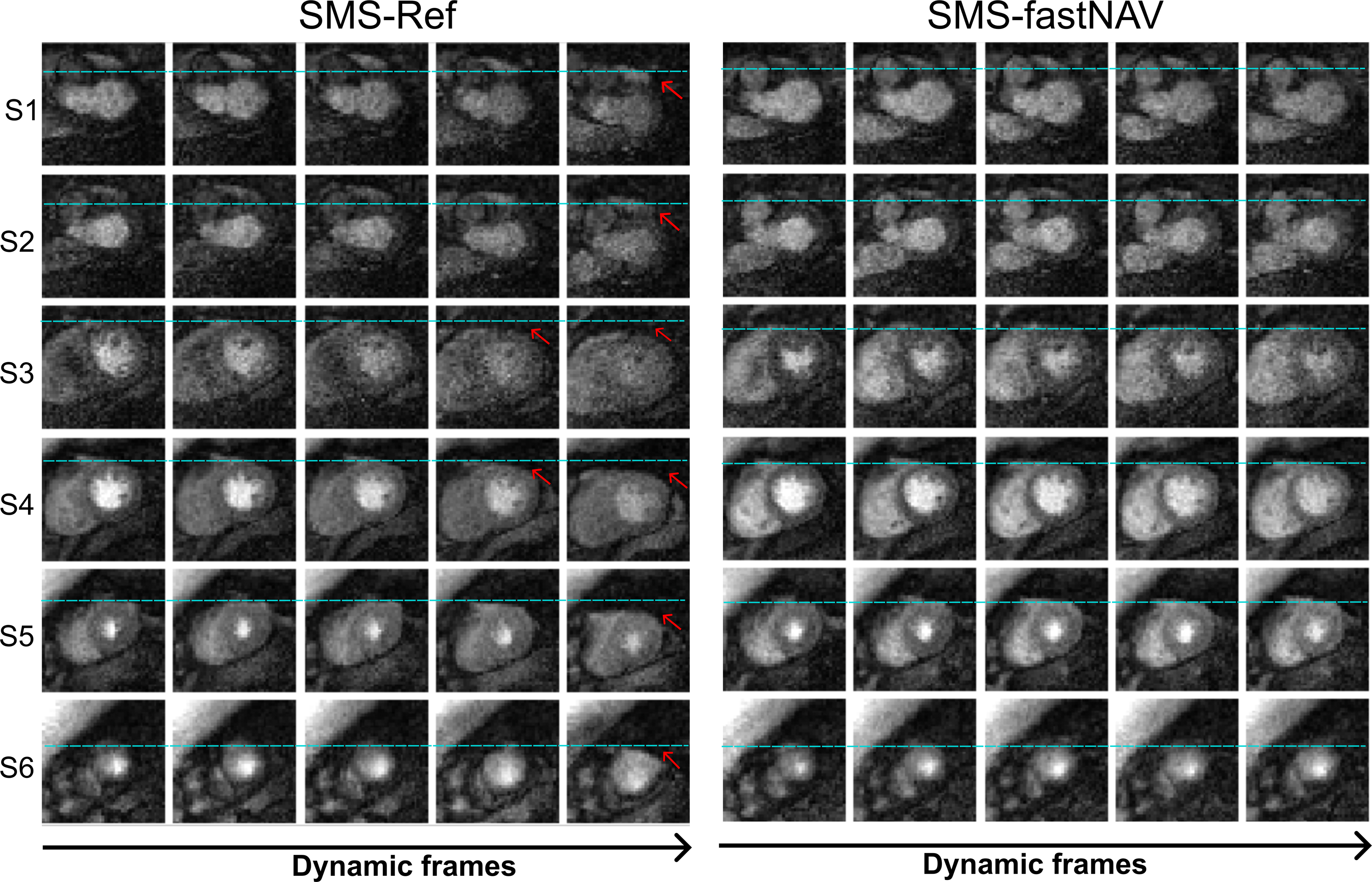

Supplement: Supplementary file 8 — Supplementary material [file mmc8.jpg]

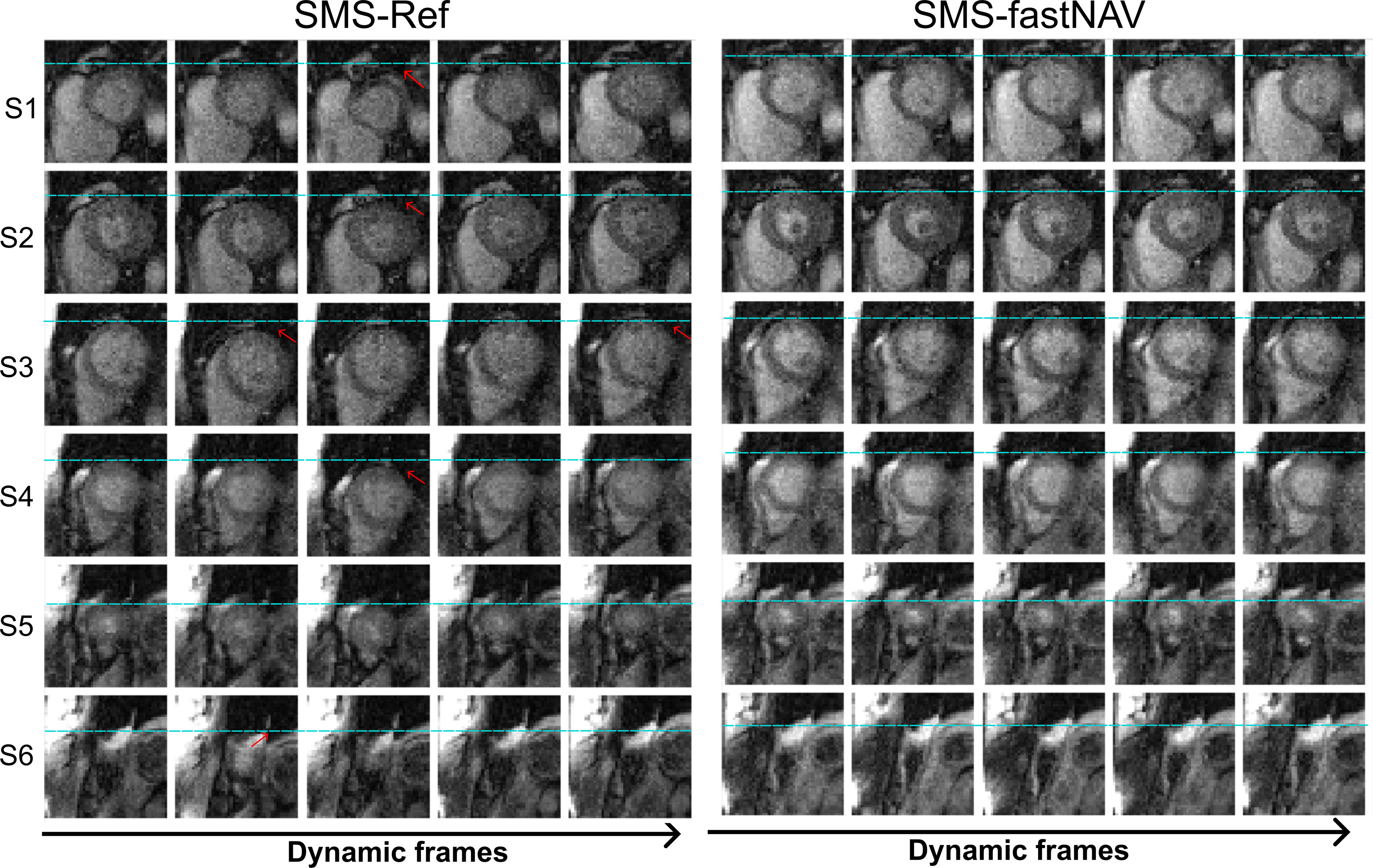

Supplement: Supplementary file 9 — Supplementary material [file mmc9.jpg]

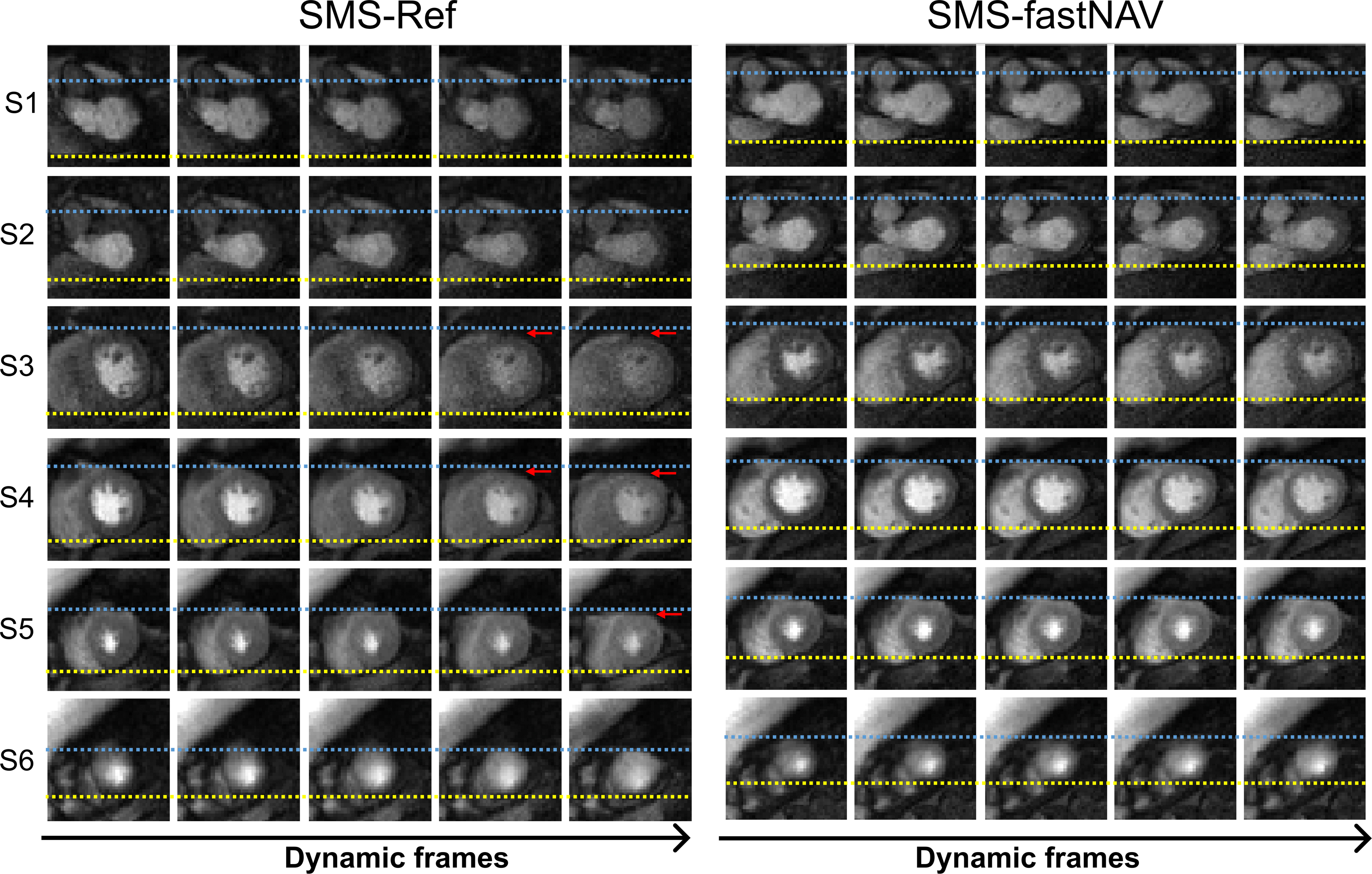

Supplement: Supplementary file 10 — Supplementary material [file mmc10.jpg]

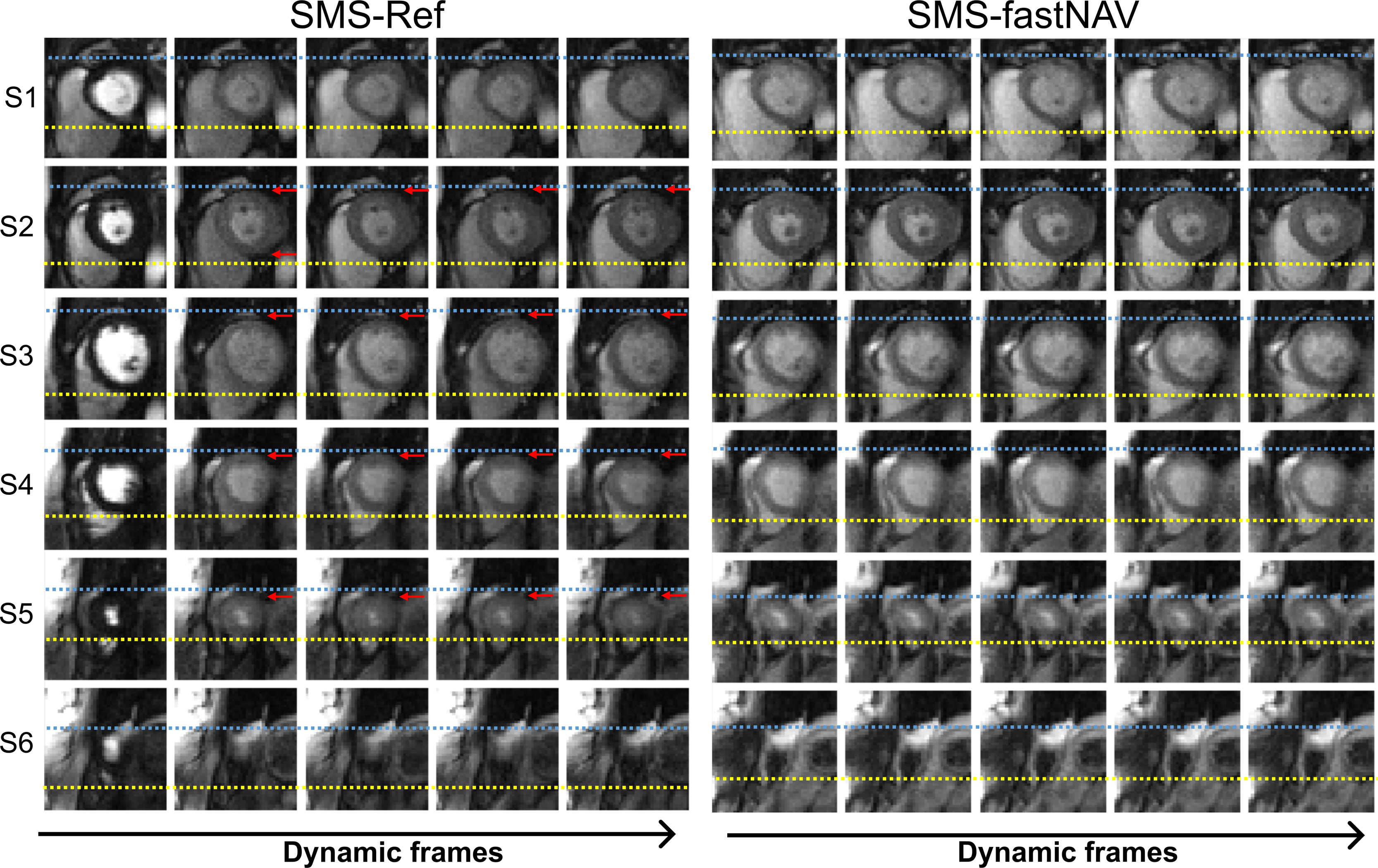

Supplement: Supplementary file 11 — Supplementary material [file mmc11.jpg]
